# Tumor Activated Cell Penetrating Peptides to Selectively Deliver Immune Modulatory Drugs

**DOI:** 10.3390/pharmaceutics13030365

**Published:** 2021-03-10

**Authors:** Dina V. Hingorani, Maria F. Camargo, Maryam A. Quraishi, Stephen R. Adams, Sunil J. Advani

**Affiliations:** 1 Department of Radiation Medicine and Applied Sciences, University of California San Diego, La Jolla, CA 92093, USA; dhingorani@ucsd.edu (D.V.H.); mcamargo@ucsd.edu (M.F.C.); mquraish@ucsd.edu (M.A.Q.); 2 Department of Pharmacology, University of California San Diego, La Jolla, CA 92093, USA; sadams@ucsd.edu; 3 Moores Cancer Center, University of California San Diego, 3855 Health Sciences Drive, La Jolla, CA 92093, USA

**Keywords:** targeted drug delivery, cell penetrating peptides, toll-like receptor ligand, matrix metalloproteinases

## Abstract

Recent advances in immunotherapy have revolutionized cancer therapy. Immunotherapies can engage the adaptive and innate arms of the immune system. Therapeutics targeting immune checkpoint inhibitors (i.e., CTLA-4; PD-1, and PD-L1) have shown efficacy for subsets of cancer patients by unleashing an adaptive antitumor immune response. Alternatively, small molecule immune modulators of the innate immune system such as toll-like receptor (TLR) agonists are being developed for cancer therapy. TLRs function as pattern recognition receptors to microbial products and are also involved in carcinogenesis. Reisquimod is a TLR 7/8 agonist that has antitumor efficacy. However, systemic delivery free resiquimod has proven to be challenging due to toxicity of nonspecific TLR 7/8 activation. Therefore, we developed a targeted peptide-drug conjugate strategy for systemic delivery of resiquimod. We designed an activatable cell penetrating peptide to deliver resiquimod specifically to the tumor tissue while avoiding normal tissues. The activatable cell penetrating peptide (ACPP) scaffold undergoes enzymatic cleavage by matrix metalloproteinases 2/9 in the extracellular matrix followed by intracellular lysosomal cathepsin B mediated release of the free resiquimod. Importantly, when conjugated to ACPP; the tumor tissue concentration of resiquimod was more than 1000-fold greater than that of surrounding non-cancerous tissue. Moreover, systemic ACPP-resiquimod delivery produced comparable therapeutic efficacy to localized free resiquimod in syngeneic murine tumors. These results highlight a precision peptide-drug conjugate delivery.

## 1. Introduction

Precision medicine in oncology requires specific target engagement within the tumor environment. Oncologic applications include site-specific delivery of a therapeutic agent for tumor kill or localizing an imaging agent for tumor delineation using carrier vehicles (i.e., nanoparticles, liposomes, antibodies, and peptides) [1]. In principle, tumor localized delivery by carriers can be accomplished through either passive or active targeting strategies. For passive targeting, generic tumor and normal tissue differences have been exploited to enhance carrier vehicle accumulation in tumors. Examples of this approach include the enhanced permeability and retention (EPR) effect and hypoxia [2,3,4,5]. Nanoparticles and liposomal based carriers have been designed to take advantage of such tissue differences to preferentially localize carrier vehicles to tumors [6,7,8].

Alternatively, active targeting approaches have been developed to specifically recognize tumor characteristics (i.e., overexpressed receptors and enzymes) that are then biochemically exploited. Intact, active delivery carriers hold the associated drug or imaging agent in a biologically inert state creating a “pro-drug”. Carrier vehicles have been engineered with chemical scaffolds that allow for the bound agent to circulate in body in an inactive, stealth mode. Upon engaging their biological target (i.e., elevated cell surface receptors or tumor microenvironment protease), they are uncloaked and deliver their payload specifically within tumors [9,10]. Examples of active targeted carriers include antibody drug conjugates (ADC) [11], cell penetrating peptide drug conjugates [12,13], disassembling polymers [14], coagulating polymers [15], sugars [16], and lectins [17]. An example of a targeted cell penetrating peptide was developed in the lab of the late Dr. Roger Y. Tsien, 2008 Nobel Prize in Chemistry [18,19]. This delivery system has been termed activatable cell penetrating peptide (ACPP). ACPPs minimally consist of a polycationic cell penetrating peptide and an autoinhibitory polyanionic peptide separated by intervening enzyme responsive cleavable peptide linker sequence. The ACPP scaffold was designed with a modular architecture to allow for versatile biomedical applications. Importantly, the termini of the ACPP probes can be conjugated to dyes or drugs for imaging and therapeutic applications. Initial studies from our labs have shown the therapeutic potential of ACPPs in cancer therapies by conjugating potent cytotoxins or radiosensitizers to ACPPs and demonstrating their direct antitumor efficacy [20,21,22].

While direct tumor cell kill has been the predominant paradigm in cancer therapy, recent excitement has centered on cancer immunotherapies that engage the immune system to attack cancers. Within the last decade, immune checkpoint inhibitors have revolutionized cancer treatment by recruiting the adaptive immune system to recognize and eradicate tumor cells. While clinical success with immunotherapy has centered on adaptive immunity, another aspect of tumor immune response involves the innate immune system [23]. Toll-like receptors (TLR) function as pattern recognition receptors to microbial products and are also involved in carcinogenesis. Intriguingly, potent TLR agonists stimulate innate immunity and have been found to have antitumor efficacy. For example, the TLR7 agonist imiquimod is topically used to treat patients with basal cell skin cancers [24]. Resiquimod or R848 is an imidazoquinoline derivative that acts as a selective agonist for the powerful immunostimulatory TLR7 and TLR8 receptors. Resiquimod was originally developed for treating skin lesions as a topical gel due to its antiviral and antimicrobial properties [25,26]. More recently, resiquimod has been found to have antitumor potential. Resiquimod triggers the release of pro-inflammatory cytokines [27] and reduces the immunosuppressive function of myeloid derived suppressor cells (MDSCs) [28]. Moreover, resiquimod polarizes macrophages from a M2 to M1-like phenotype, which is thought to promote antitumor macrophage activity [29]. Unfortunately, resiquimod’s toxicity as a free drug has made systemic delivery an issue and limited its clinical utility [30,31,32,33] [34]. As a result, research has focused on targeted delivery of resiquimod to improve pharmacodynamics and pharmacokinetics of the drug for cancer therapies [6,7].

Here we report a precision delivery approach for resiquimod using tumor targeted cell penetrating peptides by conjugating resiquimod to ACPPs. First, we validated that ACPP architecture cloaked cell penetrating peptides in murine syngeneic tumor models. While naked cell penetrating peptides are non-specifically taken up by murine tumor cells, incorporating cell penetrating peptides into the ACPP molecular scaffold blocked the inherent non-discriminatory uptake of cell penetrating peptides. Using a ratiometric ACPP with fluorescent labels, we showed that ACPPs are selectively cleaved in situ within tumors. We then successfully synthesized an ACPP-resiquimod conjugate. Importantly, we demonstrated that conjugated resiquimod released from the ACPP scaffold within tumors resulting in selective drug delivery to tumors while avoiding the surrounding, adjacent non-tumor tissue. Therapeutically, we found that systemic intravenous (i.v.) injection of ACPP-resiquimod conjugate resulted in tumor regression in the aggressive B16F10 melanoma syngeneic animal mode.

## 2. Methods

### 2.1. Synthesis of ACPP-Resiquimod Conjugate

To synthesize ACPP conjugate, resiquimod was first attached to a maleimidocaproyl-valine-citrulline-p-aminobenzyl carbamyl (MC-VC-PABC) linker. Resiquimod (10 mg, 31.8 µmol, Selleckchem, Houston, Texas, USA), MC-VC-PABC-PNP (80%, 29.5 mg, 32 µmol, Synchem, Chicago, Illinois, USA) and HOBt (1-hydroxybenzotriazole hydrate, 0.86 mg, 6.4 µmol, Sigma-Aldrich, St Louis, Missouri, USA) was dissolved in dry dimethylformamide (DMF) (100 µL, Sigma-Aldrich, St Louis, Missouri, USA) under argon gas (Ar) and kept at room temperature in the dark for five days. An additional 100 µL of DMF was added to the viscous solution and reaction continued for a further seven days when LC-MS revealed 50% reaction of resiquimod. The desired product was separated by RP-HPLC (C18 column, acetonitrile (ACN-)water-0.05% trifluoroacetic acid (TFA)) and lyophilized to a white powder identified as MC-VC-PABC-resiquimod. Yield 11.3 mg, 12.4 µmol (39%). ES-MS, found 913.4 (M+H^+^), calculated for C_46_H_60_N_10_O_10_, 913.4.

The MC-VC-PABC-resiquimod was then coupled to ACPP and cyclic arginine-glycine-aspartate (cycloRGD) attached.

H_2_N-peg8-e_9_-oPLGC(Me)AG-r_9_-c-CONH_2_ (where upper and lower case single letter amino acid codes reflect L- and D-residues, respectively, 10·TFA salt, 9.7 mg, 2 µmol; prepared as in Hingorani et al. [22]) dissolved in dry dimethylsulphoxide DMSO (100 µL, Sigma-Aldrich, St Louis, Missouri, USA) was mixed with MC-VC-PABC-resiquimod (2.4 mg, 2.6 µmol) dissolved in dry DMSO (250 µL) and N-methyl morpholine (NMM) (2.2 µL, 20 µmol, Sigma-Aldrich, St Louis, Missouri, USA) and kept at room temperature. LC-MS indicated complete reaction after 1 h to give a single product that was used without further purification, ES-MS found 580.7 (M+8H^+^), 663.5 (M+7H^+^), 773.9 (M+6H^+^), 928.5 (M+5H^+^), 1160.4 (M+4H^+^), deconvolved to 4637.5 (M+H^+^), calculated for C_193_H_319_FN_65_O_64_S_2_, 4637.3, that was used without further purification.

Cyclo (RGDfC) (2.24 mg, 3.8 µmol, Peptide international, Louisville, Kentucky, USA) dissolved in dry DMSO (100 µL) was mixed with a solution of 6-maleimidocaproic acid (2-nitro-4-sulfo) phenyl ester (Bachem; 1.09 mg, 2.5 µmol, Bachem, Torrance, California, USA) in dry DMSO (25 µL) and NMM (1.1 µL, 10 µmol). LC-MS indicated complete reaction after 30 min to yield the adduct, ES-MS found 991.4 (M^+^+H^+^), calculated for C_40_H_50_N_10_O_16_S_2,_ 991.28. This crude product was added to the reaction mix of H_2_N-peg8-e_9_-oPLGC(Me)AG-r_9_-c-(MC-VC-PABC-resiquimod)-CONH_2_ prepared above and kept at room temp until complete by LC-MS (1–2 weeks), and then was quenched with HOAc (50 µL), separated by HPLC and lyophilized to give cyclo(RGD)fc-MC-HN-peg8-e_9_-oPLGC(Me)AG-r_9_-c-(MC-VC-PABC-resiquimod-CONH_2_ a white powder. Yield, 10.25 mg (80% as salt with 9 TFA). ES-MS found 602.1 (M+9H^+^), 677.2 (M+8H^+^), 773.8 (M+7H^+^), 902.5 (M+6H^+^), 1082.9 (M+5H^+^), 1353.4 (M+4H^+^), deconvolved to 5409.3 (M+H^+^), calculated for C_227_H_364_N_74_O_74_S_3_, 5409.6 (Supplementary Figures S1 and S2).

### 2.2. Synthesis of Ratiometric ACPP, ACPP-Cy5 Conjugate and CPP-Cy5 Conjugate

Ratiometric activatable cell penetrating peptide was synthesized as previously described (Supplementary Figures S3 and S4) [35]. For the ACPP-Cy5 conjugate, 1.1 equivalent of Cy5-maleimide (MW = 778 g/mol) was coupled to the ACPP peptide, followed by acylation with cyclo(RGDfC) as described previously for ACPP-resiquimod conjugate (Supplementary Figure S5). To label r9 cell penetrating peptide, 1.1 equivalent of Cy5-maleimide was incubated with purified peptide r9-c (MW = 1526 g/mol, Biomatik, Willmington, DE, USA) with d-amino acids in dry DMSO and 10 equivalents of NMM for 1 h at room temperature. The final product was purified by RP-HPLC with a gradient of 0% Water +0.025% TFA for 10 min, followed by 10–50% Acetonitrile in water with 0.05% TFA for 20 min. ES-MS found 769.5 (M+3H^+^), deconvolved to 2305.5 (Supplementary Figure S6). The peptide was lyophilized and stored as a powder at −20 °C.

### 2.3. Cell culture Cy5 Imaging

Murine B16F10 melanoma and LL2 Lewis lung carcinoma cell lines were purchased from ATCC. MC38 colon adenocarcinoma cell line was purchased from Kerafast (Boston, MA, USA). Cell lines were grown in Dulbecco’s modified eagle’s medium (DMEM) + 10% fetal bovine serum (FBS) media. All cell lines were maintained at low passage and routinely tested for mycoplasma. For Cy5 imaging, cells were resuspended and incubated with Cy5 labeled r9 or ACPP at 1 μM in 1% FBS. The cells were incubated for 2 h under rotation and covered to be protected from light at room temperature. Cells were then spun down (5 min at 2000 rpm) and resuspended in cell media for an additional 2 h. The cells were then fixed with 4% paraformaldehyde in phosphate buffered saline (PBS), washed twice with PBS and incubated with 4′,6-diamidino-2-phenylindole (DAPI) stain (1:500). Finally, the cells were washed with PBS and mounted on slides using Fluoromount mounting media (Sigma, St Louis, MI, USA). Cells were imaged using a Keyence microscope with a 100× oil objective and high-resolution settings.

### 2.4. In Vivo Murine Fluorescent Imaging Experiments

All animal experiments were conducted in accordance with our approved protocol under the Institutional Animal Care and Use Committee (IACUC) at the University of California San Diego. Animals were purchased from Jackson Labs. Female six-week-old C57Bl6 albino mice were injected with 50,000 LL2 cells or B16F10 cells subcutaneously in both thighs using a 1:1 PBS:Matrigel mixture (Corning. Inc., Corning, NY, USA). Once tumor volumes reached approximately 100–150 mm^3^, mice were intravenously (i.v.) injected with 10 nmoles of ratiometric ACPP in 100 µL of sterile water. Ninety minutes after injection the mice were sacrificed, skin removed, and whole mice imaged using the Maestro small animal imager (CRI). The acquisition parameters were excitation filter 607/36 nm, emission filter 633 LP and exposure time of 400 ms. The in vivo images were analyzed with custom software for generating Cy5/Cy7 ratiometric pseudocolor images.

### 2.5. Confocal Imaging Of Murine Tissue Sections

LL2 tumor tissue were cryosectioned (Leica, Buffalo Grove, IL, USA) at 10 µm thickness onto Truebond glass slides VWR, Radnor, PA, USA). Tissue slides were stained with DAPI (1 mg/mL) for 10 min and then imaged on a Nikon A1 upright confocal microscope using a 20×/0.75 ∞/0.17 WD 1.0 air objective. The acquisition parameters were pixel dwell of 1.2 µs, aperture size 1.2 au, at 405 nm laser line power of 5% and gain of 80 was used, at 640 nm laser line power of 30% and gain of 100 was used (values variable depending on power of laser light source). The Nyquist feature was used to obtain images at optimal resolution resulting in an image resolution of 0.2 µm/px for 2 k sized 3 by 3 tile.

### 2.6. Tissue Drug Concentration

Female six-week-old C57Bl6 albino mice were injected with 50,000 B16F10 melanoma cells subcutaneously into both thighs using a 1:1 PBS:Matrigel. Mice were i.v. injected with 30 nmoles of ACPP-resiquimod in 100 µL sterile water and 6 h post injection mice were sacrificed. Tumor and adjacent muscle tissue were excised, weighed, and flash frozen in liquid N_2_. A mixture of 44 mL H_2_O, 44 mL acetonitrile, and 2 mL glacial acetic acid was used as the diluent. Nine times *w/v* of diluent was added to the frozen tissue. Tissues were dissociated on ice with a Sonic dismemberator Model 500 (Fisher Scientific) tissue probe homogenizer using 5 s pulse of 20% amplitude four to five times. The samples were spun down to obtain clear supernatant. For muscle tissue, addition passage through a 0.22 µm Nylon filter (Costar Spin X; purchased from Sigma, St Louis, MI, USA) was used to obtain clear supernatant. A fixed volume of tissue supernatant from each sample was injected on the LC-MS/MS (Agilent 1100 LC with MSD Trap XCT, Santa Clara, CA, USA). Each sample was run in duplicate on the LC-MS/MS to determine reproducibility of integrated ion current for each sample. A gradient of 5–90% of acetonitrile in water with 0.05% TFA in 20 min was used with the Luna 5µm C18(2) 100 Å, 250 × 4.6 mm column and MS parameters were set for fragmentation of m/z = 315. The ion current for m/z 251.0 (315) was extracted and integrated over retention time of 12 to 12.4 min. A standard calibration was generated by making a series of known concentrations of resiquimod (0, 5, 10, 30, 80, and 150 nM) using supernatant from control tissue samples as the diluent. Concentration of unknown samples were then calculated (Graphpad PRISM).

### 2.7. In Vivo Murine Therapy Experiments

Female six-week-old C57Bl6 albino mice were injected with 50,000 B16F10 melanoma cells subcutaneously in both thighs using a 1:1 mixture of PBS:Matrigel. The tumors were palpable five days after implant at which point treatment was initiated. Due to the rapid growth of B16F10 tumor in mice, dosing began when tumors reached an average size of 30 ± 13 mm^3^. Animals were divided into four treatment groups: control, intratumoral (i.t.) resiquimod (30 nmoles per tumor), i.t. ACPP-resiquimod (30 nmoles per tumor), or i.v. ACPP-resiquimod (60 nmoles per mouse). Mice were given free drug or ACPP conjugated on day 5 and day 9. Each treatment group had five mice with 10 tumors. Tumor volumes were measured bi-weekly and mice weighed weekly. Mice were sacrificed when the tumor size reached 1500 mm^3^.

## 3. Results

### 3.1. Tumor Targeted Activatable Cell Penetrating Peptides

**Figure 1:**
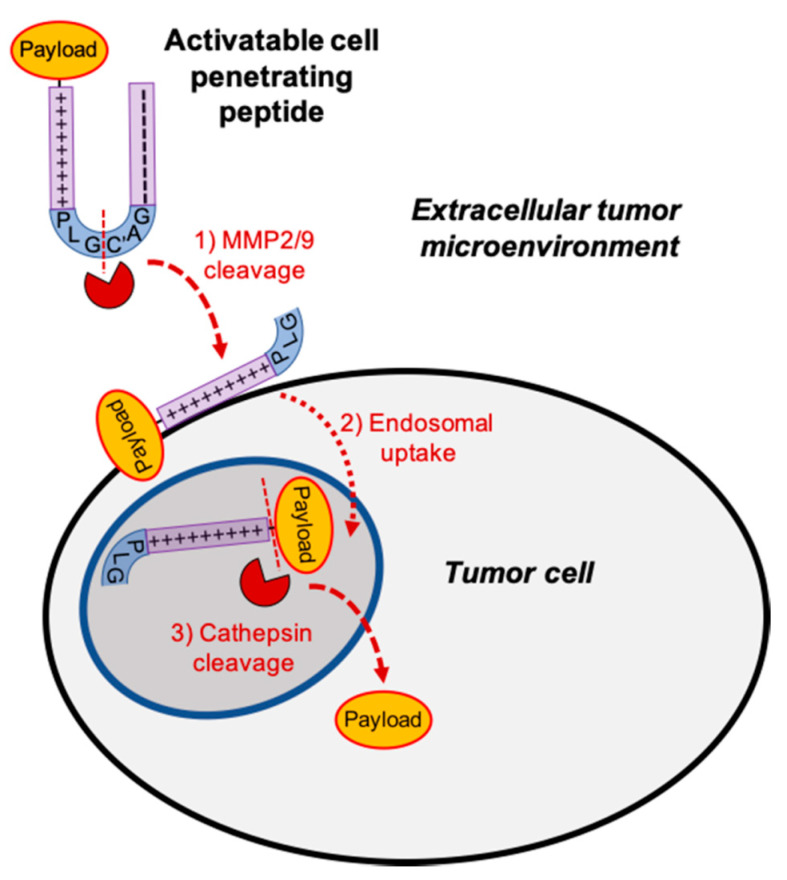
Tumor targeted activatable cell penetrating peptides. Model for activatable cell penetrating peptide tumor localization. Polycationic cell penetrating peptide (+) and autoinhibitory polyanionic peptide (−) are connected by a metalloproteinase (MMP) 2/9 sensitive peptide linker (PLGC(Me)AG).

Tumor specific drug delivery requires three key steps: (1) targeted delivery to the tumor following systemic administration; (2) intracellular cell uptake upon arrival at the tumor; and (3) pharmacologically active drug once within the cell. Activatable cell penetrating peptides (ACPP) were rationally designed to provide a solution to all three of these requirements for tumor targeted drug delivery. ACPP consist of three regions: a polycationic cell penetrating peptide (CPP), a polyanionic autoinhibitory domain, and a flexible protease sensitive peptide linker (Figure 1). The polycationic cell penetrating peptide consists of nine repeats of d-arginine (r9) and the polyanionic autoinhibitory peptide is a nine amino acid repeat of d-glutamic acid (e9). To improve stability and protect ACPP from degradation by endogenous enzymes while in systemic circulation, the cell penetrating peptide of ACPP consists of d-amino acids [36]. For tumor targeted delivery, a PLGC(Me)AG peptide linker was inserted between the r9 and e9 polypeptides. The PLGC(Me)AG sequence is cleaved by gelatinase matrix metalloproteinase 2 and 9 (MMP 2/9). MMP 2/9 activity is abundant in extracellular tumor microenvironments conferring tumor targeted capability to ACPPs. While ACPP is systemically circulating, the polycationic CPP is neutralized by the proximity to the polyanionic peptide and essentially inert. Following MMP 2/9 cleavage of the PLGC(Me)AG linker within tumor microenvironments, the ACPP is “activated” with release of the r9 CPP. Electrostatic interaction between the polycationic CPP and cell membrane results in tumor cell surface adhesion followed by endosomal uptake [19]. For therapeutic applications, drugs can be conjugated to the polycationic cell penetrating peptide of ACPP though a maleimidocaproyl-valine-citrulline-p-aminobenzyl carbamyl linker (MC-VC-PABC) [20]. The MC-VC-PABC linker was chosen for drug attachment since it was used in a clinically approved ADC [11]. Moreover, the MC-VC-PABC linker is cleaved by cathepsin B within endolysosomes to intracellularly release pharmacologically active free drug in a self-immolative reaction. MMP-2/9 targeted ACPP was also constructed with a cyclic RGD peptide on the d-glutamic acid arm (e9) for additional targeting to the α_v_β_3_ integrin that is found on the membranes of many cancer cells [20]. It has been reported that α_v_β_3_ integrins are associated with MMP-2/9 activity [37]. Thus, our ACPP tumor drug delivery vehicles provide a solution for tumor-targeted localization (MMP 2/9 cleavable linker), intracellular delivery (r9 CPP), and free drug release (cathepsin B cleavable linker). The inherent versatility of ACPP’s architecture allows for both therapeutic and diagnostic oncologic applications.

### 3.2. Intact ACPP Cloak Polycationic Cell Penetrating Peptides

**Figure 2:**
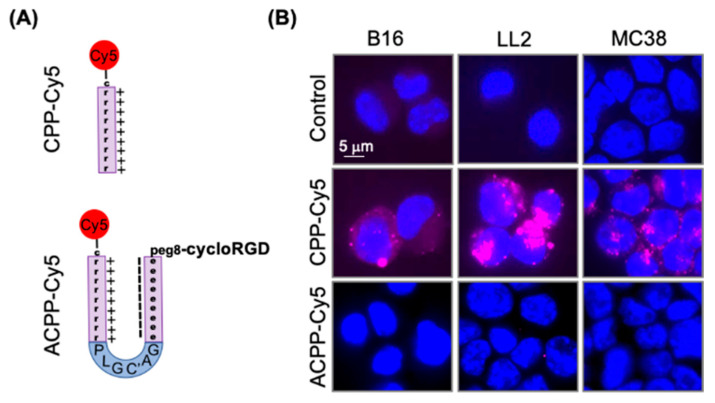
Activatable cell penetrating peptide (ACPP) scaffold blocks cell penetrating peptide (CPP) uptake by murine tumor cells. (**A**) Structural representation of Cy5 labeled CPP and ACPP. (**B**) Murine cancer cells exposed to Cy5 labeled CPP and ACPP probes. Cells imaged for Cy5 fluorescence (Magenta). Nuclei stained with DAPI (Blue).

We first determined the ability of intact ACPP to block cell penetrating peptide dependent uptake in murine tumor cell lines. Polycationic cell penetrating peptide consisting of nine d-arginine repeats was labeled with Cy5 dye (Figure 2A, Supplementary Figure S6). Murine B16, LL2, and MC38 cells were exposed to 1 μM Cy5 labeled r9 and visualized by fluorescent microscopy (Figure 2B). The r9 polycationic cell penetrating peptide was readily taken up by murine cells in 2 h. Similarly, we Cy5 labeled an ACPP with a cloaked r9 polycationic cell penetrating peptide (Supplementary Figure S5). Importantly, the ACPP scaffold prevented the associated r9 polycationic cell penetrating peptide from binding to murine cells (Figure 2B).

### 3.3. Cleavage of PLGC(Me)AG Targeted ACPPs in Live Mice

**Figure 3:**
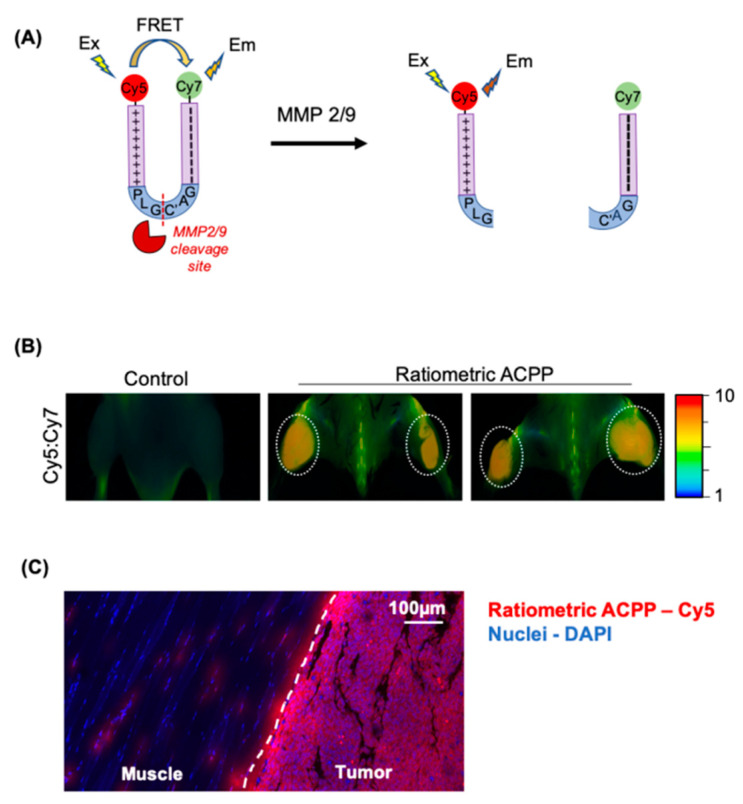
ACPPs are cleaved in situ within murine tumors. (**A**) Structural representation of ratiometric activatable cell penetrating peptide probe with intervening MMP-2/9 protease sensitive amino acid sequence. (**B**) Mice with subcutaneous syngeneic LL2 tumors tail vein injected with 10 nanomoles ratiometric ACPP. In situ mouse imaging for Cy5:Cy7 emission ratio shown. Cy5:Cy7 emission ratio pseudocolor scale bar shown on far right. (**C**) Confocal imaging of tissue from LL2 tumor bearing mice injected with ratiometric ACPP. Tissue section containing tumor and adjacent muscle imaged for Cy5 (Red). Nuclei DAPI stained (Blue).

ACPP cleavage and release of its polycationic cell penetrating peptide requires tumor microenvironment proteases. To determine if ACPP cleavage occurs in situ within tumors, we synthesized a ratiometric ACPP where a Cy5 dye is attached to the poly d-arginine arm and a Cy7 dye is attached to the poly d-glutamate arm (Figure 3A, Supplementary Figure S4). In intact ACPP, the Cy5 and Cy7 dyes are in close spatial proximity allowing for efficient fluorescence resonance energy transfer (FRET) from Cy5 to Cy7, resulting in a low ratio of the Cy5:Cy7 emissions following excitation of Cy5 at 640 nm. Upon ACPP cleavage and the resultant spatial separation of the Cy5 and Cy7, the FRET is lost and Cy5 emission increases 10 fold relative to Cy7 [35]. Mice bearing bilateral LL2 tumors in the thighs were i.v. injected with 10 nmoles of ratiometric ACPP (Figure 3B). Mice were then imaged at 90 min post injection. Murine syngeneic tumors injected with ratiometric ACPP had an elevated Cy5:Cy7 emission ratio within tumors while the non-tumor tissue showed significantly lower Cy5:Cy7 emission ratio. Fluorescence imaging with RACPP in B16F10 flank tumors in mice was challenging due to melanin content of the cells (Supplementary Figure S7). To microscopically examine Cy5 localization, cryosections of LL2 tumors with surrounding muscle were fixed and imaged by confocal microscopy (Figure 3C). Cy5 imaging validated that ACPP spatially localized within tumors while avoiding the adjacent normal tissue muscle.

### 3.4. Synthesis of ACPP Resiquimod Conjugate

**Figure 4:**
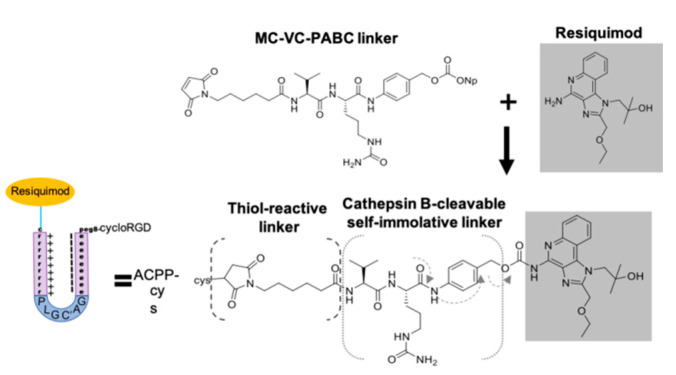
Conjugating resiquimod to ACPP. Chemical synthesis attaches resiquimod to a MC-VC-PABC linker.

To test the ability of ACPP to target immune modulating drug to tumors, we attached resiquimod to ACPP (Figure 4, Supplementary Figure S2). First, we coupled resiquimod to the MC-VC-PABC linker by reacting MC-Val↓Cit-PAB-PNP linker with the primary amine on resiquimod to form the stable carbamate with loss of para-nitrophenol. The amide bond between valine-citrulline residues can be cleaved by endosomal cathepsin B enzyme in the cytosol of cells [38]. This triggers a self-immolative reaction resulting in spontaneous formation of aza p-quinone methide, carbon dioxide, and unmodified free resiquimod (Figure 4).

The maleimide moiety on the linker-drug conjugate is then reacted via a Micheal addition to the C-terminal cysteine sulfhydryl of the ACPP scaffold H_2_N-peg8-e_9_-oPLGC(Me)AG-r_9_-c-CONH_2_ so that upon MMP cleavage and endocytosis of the r9 fragment the drug payload enters into the cytoplasm of target cells. Cyclo(RGDfc) was conjugated to an bifunctional amine-reactive MC linker via similar maleimide-cysteine chemistry. The 2-nitrophenylsulfonate active ester then allowed mild acylation of N-terminal amine of H_2_N-peg8-e_9_-oPLGC(Me)AG-r_9_-c-(MC-VC-PABC-resiquimod)-CONH_2_ to form the ACPP-resiquimod conjugate.

### 3.5. ACPP-Resiquimod Biodistribution

**Figure 5:**
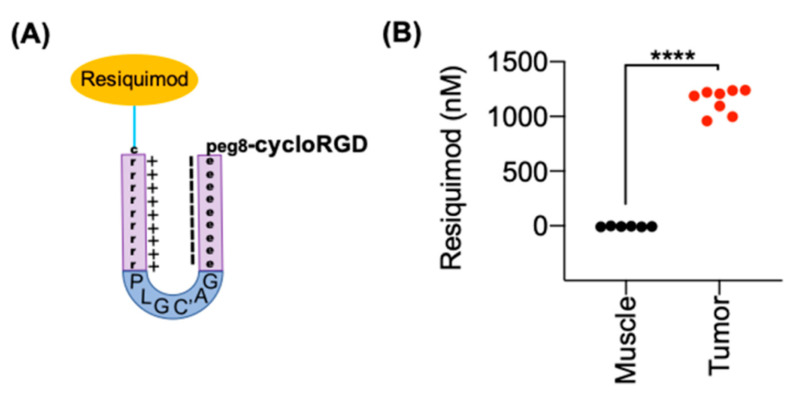
ACPP selectively delivers resiquimod to tumor tissue. (**A**) Structural representation of resiquimod conjugated to ACPP. (**B**) Resiquimod drug concentrations within tissue. Mice with B16 tumors intravenously (i.v.) injected with ACPP-resiquimod. Tumor and muscle drug concentrations quantitated by LC-MS/MS. Individual tissue concentrations plotted from muscle and tumor. **** *p* < 0.0001.

Next, we determined if ACPP-resiquimod conjugate selectively released free drug within tumors. We used B16F10 melanoma tumors since they are routinely used to evaluate cancer immunotherapies in mice. Mice with B16F10 melanoma tumors were i.v. injected with 30 nmoles of ACPP-resiquimod (Figure 5A). Tumor and adjacent normal muscle tissues were harvested. Resiquimod drug concentrations were determined by LC-MS/MS analysis (Figure 5B). Tumor tissues had significantly higher resiquimod drug concentrations of 1142.3 ± 111.7 nM compared to −5.8 ± 3.5 nM for the peri-tumoral muscle tissue indicating that the ACPP-resiquimod specifically accumulated within tumor tissues. The tissue dependent cleavage of ACPP-resiquimod resulted in retention of the fragment r9-resiquimod, which undergoes endocytosis and further cleavage by cathepsins to release the free resiquimod. Importantly, normal tissue accumulation of resiquimod in the adjacent non-cancerous muscle was negligible by sensitive mass spectrometry based quantitative technique.

### 3.6. Antitumor Efficacy of ACPP-Resiquimod

**Figure 6:**
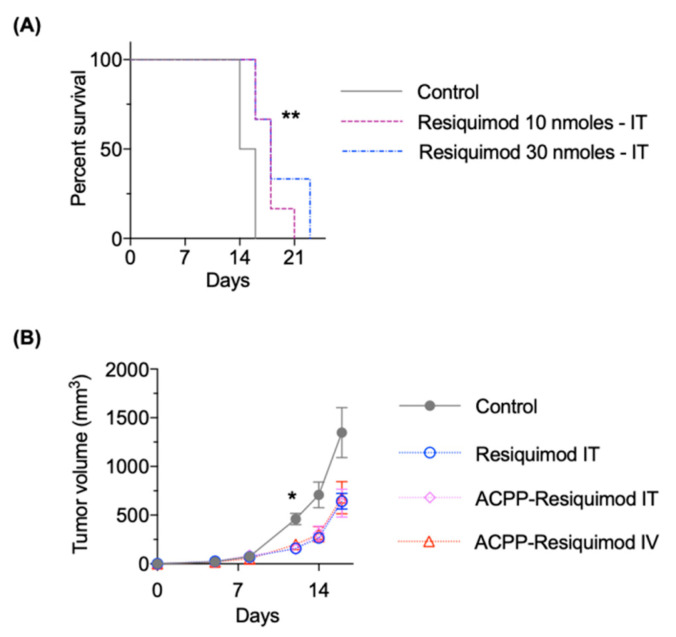
Antitumor efficacy of ACPP conjugated resiquimod. (**A**) Mice with B16 tumors intra-tumorally (IT) injected with free resiquimod. Mouse survival plotted and statistical significances calculated using Log-rank (Mantel–Cox) test. (**B**) Mice with B16 tumors treated IT with free resiquimod or ACPP conjugated resiquimod given IT or intravenously (IV). Tumors measured twice a week and plotted as mean tumor volume ± SEM. Statistical significances calculated using two-way ANOVA. * *p* < 0.05, ** *p* < 0.01.

Finally, we determined the antitumor efficacy of ACPP-resiquimod conjugate. We first performed dose response studies with unconjugated resiquimod by i.t. injections in mice with B16F10 melanoma tumors. Mice treated with 30 nmoles of free resiquimod had statistically improved survival compared mice treated with 10 nmoles (Figure 6A). Next, we tested the antitumor efficacy of free resiquimod compared to the ACPP conjugate (Figure 6B). Free resiquimod was given by i.t. injection. ACPP-resiquimod was given either i.t. or i.v. The total amount of drug injected per animal was held constant to allow for comparison of i.t. vs i.v. delivery. Importantly, mice treated with resiquimod as free drug or ACPP conjugate had improved tumor control compared to control untreated mouse tumors. Interestingly, the mode of administration of ACPP-resiquimod (i.e., i.t. vs i.v.) both produced similar antitumor responses. Taken together, the data suggests that ACPP-resiquimod appears to be as efficacious as free resiquimod and can be delivered systemically.

## 4. Discussion

In this series of studies, we have described an active targeting scaffold to deliver an immune modulator for cancer therapy and evaluated it in syngeneic murine tumors. To investigate the “stickiness” or adherent properties of the poly d-arginine arm of ACPP in murine cells, we labeled r9 polypeptide with Cy5. The r9 scaffold is made of guanidinium side chains, which typically have a pKa of 12.4 and are, therefore, positively charged. This property allows the r9 molecule to non-specifically bind to the negatively charged phospholipid bilayer of any cell within its vicinity. The molecule undergoes endocytosis making it an excellent delivery system for therapeutic molecules. To overcome the nonspecific cell uptake of polycationic cell penetrating peptides, the ACPP scaffold was developed to utilize tissue endopeptidases for targeted cell penetrating peptide delivery. For tumor targeting, the intervening cleavable peptide linker sequence of ACPP was designed to be cleaved by MMP 2 and 9 gelatinases, since they are often present in the tumor extracellular matrix. MMP 2 and 9 are upregulated in cancers where they promote metastasis by mediating extracellular matrix degradation, which allows for cell migration [39,40]. Interestingly, elevated levels of MMPs 2/9 are associated with a poor prognosis in cancer patients. This suggests ACPPs can target drug delivery to the most aggressive and therapeutically resistant areas of tumors.

The therapeutic payload for ACPPs is conjugated to the r9 arm through a linker in a similar manner used in antibody drug conjugates (ADCs). A variety of linkers have been developed to attach drugs to antibodies for clinically approved ADCs. Non-cleavable linkers rely solely on lysosomal degradation to free the drug. Such a linker has been used in Trastuzumab-emtansine (Kadcyla) [41]. An advantage of this linker is that it generates charged metabolites, which then decrease cell escape [41]. However, the metabolites from non-cleavable linkers make quantitation of drug release difficult and may decrease the potency of the drug payload. For these reasons, we have used the cleavable MC-VC-PABC linker to attach drug payloads to ACPPs. The MC-VC-PABC linker is used in the clinically approved ADC brentuximab vedotin (Adcetris) [38]. The MC-VC-PABC linker was designed such that it undergoes self-immolation following cleavage of its valine-citrulline dipeptide by cathepsin B within endolysosomes [42]. This then results in the predictable release of free active drug from the linker. Importantly, this allows for measuring tissue drug levels. Importantly, we found a dramatic increase in the tumor to muscle resiquimod concentration in mice systemically injected with ACPP-resiquimod conjugate. Negative values of concentration for some peri-tumoral tissue samples are a consequence of elevated background noise from the calibration samples prepared in tumor tissue supernatant. Negligible drug concentration in the adjacent peri-tumoral muscle also suggests that off-site toxicity from drugs can be minimized by ACPP conjugation.

ACPPs can also serve as a tool to study the in situ activity of extracellular proteases. A ratiometric ACPP was developed by attaching Cy5 and Cy7 fluorescent dyes to polycationic and polyanionic arms of ACPP, respectively. Cy5 serves as the donor fluorophore and Cy7 serves as the acceptor fluorophore to offer an optimal combination of long wavelength excitation, stable dye combination and usable dynamic range for preclinical validation of this technology. Translationally, the superior sensitivity and specificity of ratiometric ACPPs for highlighting the margins of tumors has been demonstrated [43].

An advantage of our tumor targeted ACPPs is that they allow us to engage antitumor immune responses and evaluate therapeutic contribution of immune modulating drugs. In contrast to ADC based drug delivery, the r9 polycationic cell penetrating peptide of ACPP can be taken up by both human and murine tumor cell lines. The immune system has both positive and negative regulators to fine tune its responses to antigen challenge. While most clinical excitement has focused on “removing the brakes”, i.e., immune checkpoint blockers, activation of the immune system (“pushing the gas”) is a complimentary strategy. TLR agonists are potent stimulators of the innate immunity. However, side effects from systemic administration of TLR7 agonists limits their use in non-dermatologic cancers. As a solution to this, we conjugated resiquimod to ACPP. Importantly, our initial tumor response data demonstrated that systemically administered ACPP-resiquimod by i.v. injection achieved similar tumor control to direct i.t. free resiquimod injection. Previous literature has described the limitations of delivery i.v. resiquimod as a free drug. Active research efforts are ongoing to deliver resiquimod systemically by either cloaking the drug or delivering the free drug by direct injection to the tumor tissue [30,31,32,33] and has so far only been approved for topical application on skin. Intravenous delivery may prove to be favorable for dosing certain cancers that prove to be inaccessible or challenging for direct injection. In addition, intravenous delivery can allow drug to reach metastatic sites. While our initial data is suggestive of antitumor control and localized drug delivery with ACPP, further work needs to be done to compare the toxicity profile and levels of immune response from intravenous delivery of free resiquimod and ACPP-resiquimod. In addition, it will be useful to investigate how ACPP-resiquimod can be integrated into standard cancer therapies, i.e., chemotherapy and radiotherapy. Finally, the modular nature of ACPPs can allow for conjugation of other immune modulatory drugs. We have initially focused on resiquimod since structurally it has an amine for peptide conjugation to ACPP. As peptide linker technology advances, other small molecule immune modulators can be developed for tumor targeted cell penetrating peptide drug delivery.

## Data Availability

Data will be made available on a reasonable request.

## Supplementary Materials

The following are available online at https://www.mdpi.com/1999-4923/13/3/365/s1, Figure S1: Synthesis scheme for cRGD-ACPP-resiquimod. Figure S2: Chemical structure of cRGD-ACPP-resiquimod. Figure S3: Synthesis scheme for ratiometric ACPP. Figure S4: Chemical structure of ratiometric ACPP. Figure S5: Chemical structure of ACPP-Cy5 conjugate. Figure S6. Chemical structure of CPP-Cy5. Figure S7. ACPPs in situ cleavage within murine tumors. (A) Mice with subcutaneous syngeneic B16F10 murine melanoma tumors tail vein injected with 10 nanomoles ratiometric ACPP. In situ mouse imaging for Cy5:Cy7 emission ratio shown. Cy5:Cy7 emission ratio (B) Ex-vivo tumor tissue excised from animal and imaged for Cy5:Cy7 emission ratio. Pseudocolor scale bar shown on far right for both images.
