# Supplementary material for: Tumor Activated Cell Penetrating Peptides to Selectively Deliver Immune Modulatory Drugs
Source: Pharmaceutics. 2021 Mar 10;13(3):365. doi: 10.3390/pharmaceutics13030365 (PMC8000974; doi:10.3390/pharmaceutics13030365)
Supplement: Supplementary file 1 [file pharmaceutics-13-00365-s001.pdf]

pharmaceutics

MDPI

Supplementary Materials: Tumor Activated Cell Penetrating Peptides to Selectively Deliver Immune Modulatory Drugs

Dina V Hingorani, Maria F Camargo, Maryam A Quraishi, Stephen R Adams and Sunil J Advani *

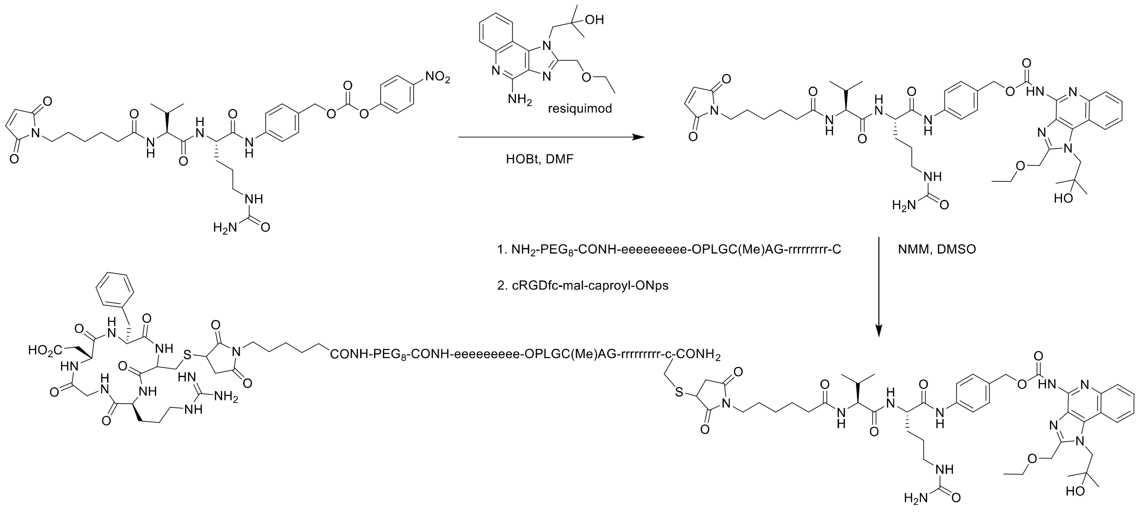

Figure S1. Synthesis scheme for cRGD-ACPP-resiquimod.

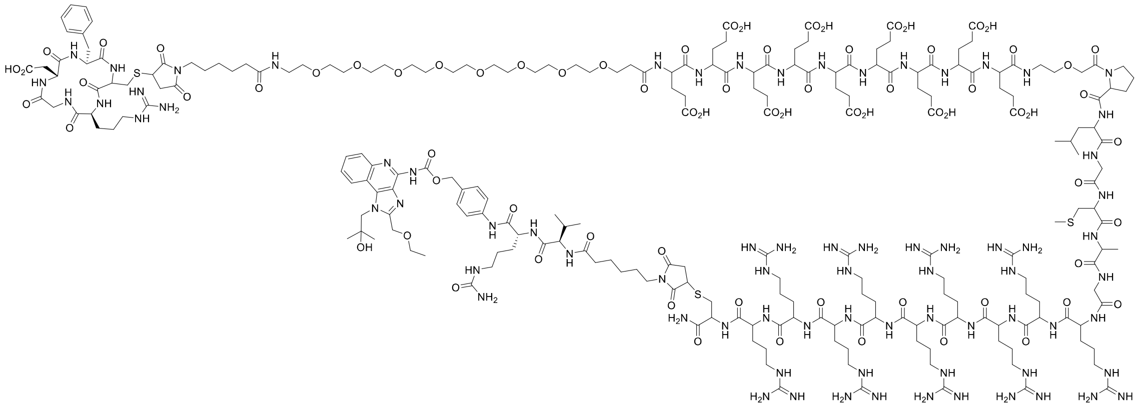

Figure S2. Chemical structure of cRGD-ACPP-resiquimod.

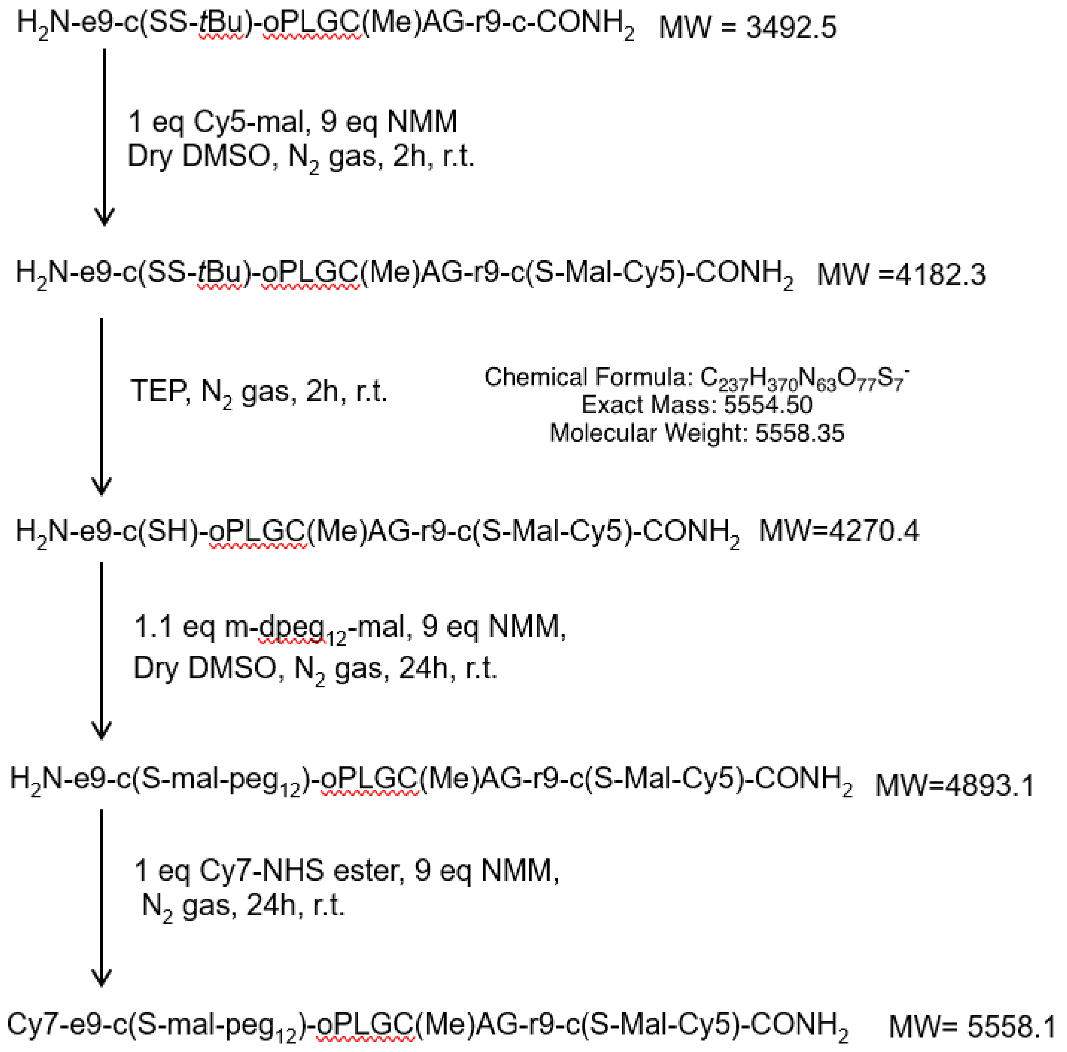

Figure S3. Synthesis scheme for ratiometric ACPP.

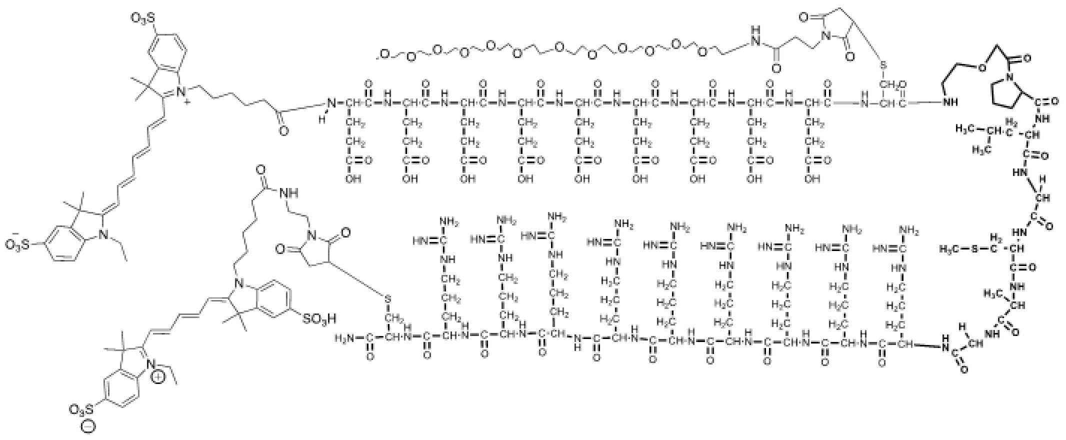

Figure S4. Chemical structure of ratiometric ACPP.

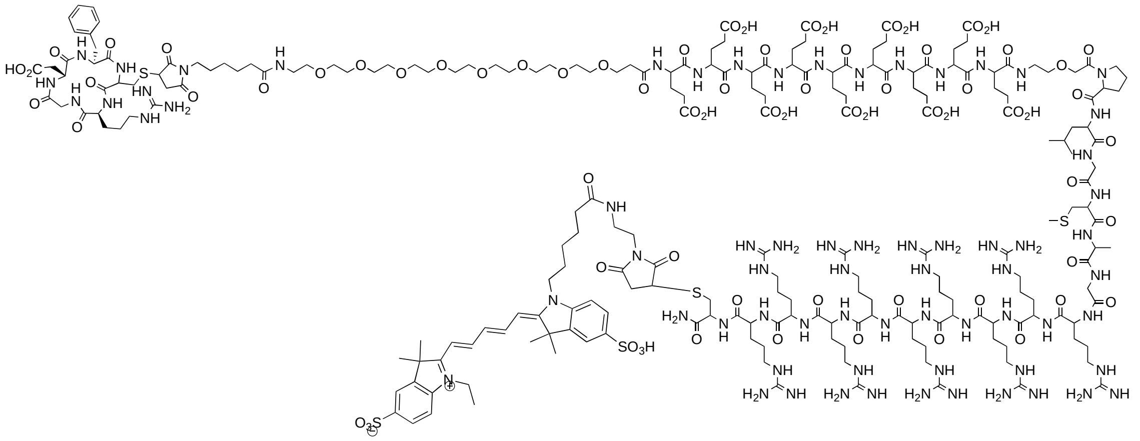

$C_{220}H_{350}N_{68}O_{73}S_5$
Exact Mass: 5272.44
Mol. Wt.: 5275.87
C, 50.08; H, 6.69; N, 18.05; O, 22.14; S, 3.04

Figure S5. Chemical structure of ACPP-Cy5 conjugate.

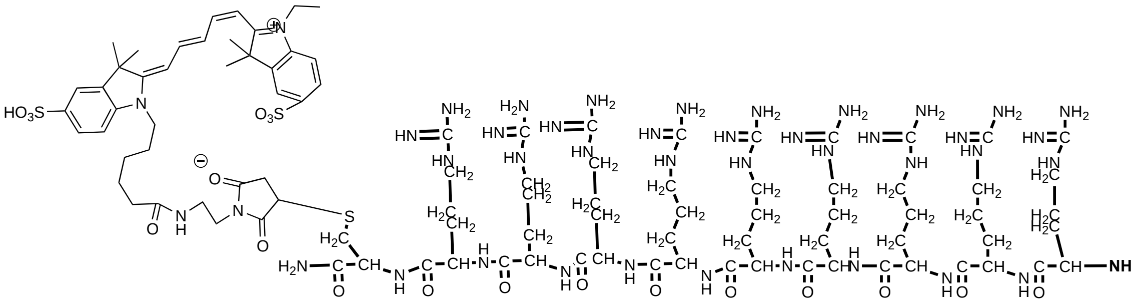

Chemical Formula: $C_{96}H_{161}N_{42}O_{19}S_3^-$
Exact Mass: 2302.21
Molecular Weight: 2303.80
Elemental Analysis: C, 50.05; H, 7.04; N, 25.54; O, 13.19; S, 4.17

Figure S6. Chemical structure of CPP-Cy5.

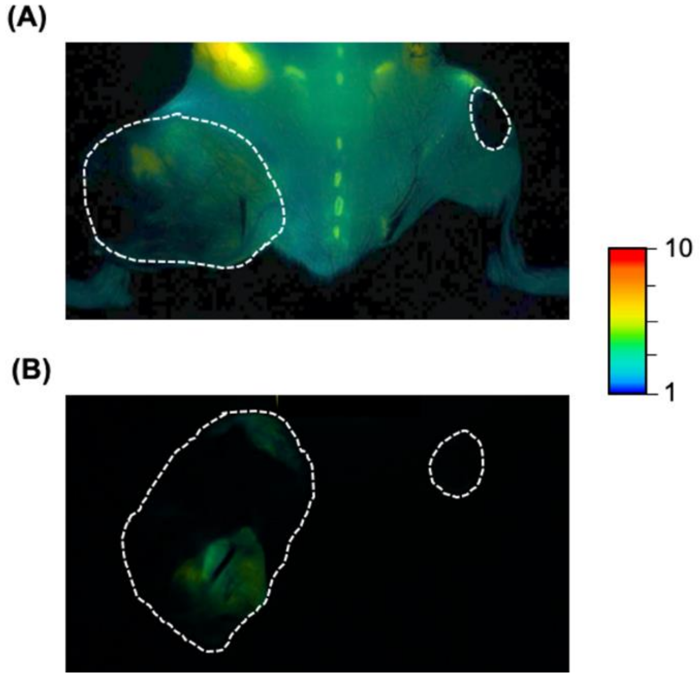

Figure 7. ACPPs in situ cleavage within murine tumors. **(A)** Mice with subcutaneous syngeneic B16F10 murine melanoma tumors tail vein injected with 10 nanomoles ratiometric ACPP. In situ mouse imaging for Cy5:Cy7 emission ratio shown. Cy5:Cy7 emission ratio **(B)** Ex-vivo tumor tissue excised from animal and imaged for Cy5:Cy7 emission ratio. Pseudocolor scale bar shown on far right for both images.
